# *GmSAUR46b* Integrates Light Signals to Regulate Leaf Midrib Thickness and Stem Trichome Density in Soybean

**DOI:** 10.3390/ijms26189200

**Published:** 2025-09-20

**Authors:** Xiao Li, Bei Liu, Yunhua Yang, Han Gou, Huan Du, Yuhao Chen, Huakun Yu, Jinming Zhao, Fengjie Yuan

**Affiliations:** 1 Xianghu Laboratory, Hangzhou 311200, China; lixiao_xiaoli@163.com (X.L.); liubei@xhlab.ac.cn (B.L.); yangyunhhua@126.com (Y.Y.); gouhan0603@163.com (H.G.); huandu504@126.com (H.D.); chenyuhao009@163.com (Y.C.); yuhuakun516@163.com (H.Y.); 2 Institute of Crops and Nuclear Technology Utilization, Zhejiang Academy of Agricultural Sciences, Hangzhou 310021, China; 3 College of Agriculture, Nanjing Agricultural University, Nanjing 210095, China

**Keywords:** *Glycine max*, *GmSAUR46b*, light response, hormone signaling, transcriptomics, leaf midrib, stem trichomes

## Abstract

Soybean (*Glycine max* (L.) Merr.) is a vital crop for the global supply of protein and oil, with its growth and development being regulated by genetic, hormonal, and environmental factors, particularly light and hormone signaling. The *Small Auxin-Up RNA* (*SAUR*) gene family plays a crucial role in plant growth regulation; however, the molecular mechanisms by which *GmSAUR46* integrates photosynthesis and hormonal networks in soybean remain unclear. In this study, we focused on *GmSAUR46b* (*Glyma.19G182600.1*) and employed CRISPR/Cas9-mediated knockout and 35S-driven overexpression lines, alongside wild-type soybean (cv. Williams 82), to investigate its function. RNA sequencing (RNA-Seq) was conducted on shoot apical meristems, stems, and leaves at three developmental stages (V1, V2, V3), followed by transcriptomic analyses, including differential gene expression (DEG) identification and functional enrichment (GO, KEGG, KOG). Anatomical studies using paraffin sectioning and scanning electron microscopy (SEM) assessed the leaf midrib thickness and stem trichome density under varying light conditions. The transcriptomic results revealed DEGs enriched in pathways related to cell wall metabolism, hormone response, and photosynthesis. Anatomical analyses demonstrated that *GmSAUR46b* specifically regulates the leaf midrib thickness and stem trichome density in a light-dependent manner: under shade, the overexpression lines exhibited increased midrib thickness and trichome density, whereas the knockout lines showed reduced trichome density. Additionally, novel transcripts associated with stress resistance, hormone metabolism, and photosynthesis were identified, expanding the known soybean gene repertoire. Collectively, *GmSAUR46b* functions as a central hub integrating light signals with hormone and cell wall pathways to modulate soybean growth, particularly leaf and stem traits. This study advances understanding of *SAUR* gene function in soybean and provides valuable insights for molecular breeding aimed at improving adaptability and yield under diverse environmental conditions.

## 1. Introduction

Soybean (*Glycine max* (L.) Merr.) is one of the most economically important leguminous crops worldwide, serving as a primary source of plant-based protein and vegetable oil for both human consumption and animal feed [1]. Its growth and development are tightly regulated by a complex interplay of genetic [2], hormonal, and environmental factors. Among these, light signaling and hormone-mediated pathways play pivotal roles in coordinating key agronomic traits such as morphogenesis [3,4,5,6,7,8,9,10], photosynthesis [11,12,13], leaf morphology [14,15,16,17], trichome development, and defense responses [18,19,20]. Understanding the molecular mechanisms underlying these regulatory networks is essential for optimizing soybean yields and adaptability under diverse environmental conditions.

The *Small Auxin-Up RNA* (*SAUR*) gene family, a large group of early auxin-responsive genes, has emerged as a central regulator of plant growth and development [21,22,23,24,25,26]. Members of this family are widely implicated in mediating cell elongation and expansion, such as hypocotyl elongation [17,27,28,29,30,31,32,33,34,35,36,37]; plant height [38,39]; organ morphogenesis, including leaf senescence, root development, stamen filament elongation, apical hook development, and hypocotyl elongation [37,40,41,42,43,44,45]; hormone signal transduction [46,47,48,49]; light response [50,51]; and responses to both biotic and abiotic stresses [52,53,54,55]. For instance, in *Arabidopsis thaliana*, constitutive expression of the *AtSAUR19* gene in tomato results in hypocotyl elongation that is independent of auxin [35]. A group of PP2C.D phosphatases localized to the plasma membrane exerts a negative regulatory effect on SAUR-mediated cell expansion [27]. Additionally, SAUR proteins and PP2C.D phosphatases modulate H^+^-ATPases and K^+^ channels, thereby governing stomatal movements [56]. In grape (*Vitis vinifera* L.), SAUR041 is a candidate regulator of berry size [57].

Notably, the regulation of *SAUR* genes exhibits remarkable diversity [26]. Their expression can be intricately modulated by various hormones, including auxin [58,59], cytokinin [59], gibberellic acid (GA) [60,61], brassinosteroids [59,62,63,64], ethylene [65], abscisic acid (ABA) [66,67], and jasmonic acid (JA) [67]. Additionally, environmental cues such as light [51,59,68,69], cold [70,71], drought [71,72,73], high temperature [74], and high salinity [71,73] also significantly influence *SAUR* expression across different plant species. Despite the identification of GmSAUR as a potential regulator in soybean [8], the molecular mechanisms underlying its integration of photosynthesis and hormonal networks remain elusive.

In this study, we focused on a candidate gene, *GmSAUR46b* (*Glyma.19G182600.1*), a member of the *GmSAUR* gene family in soybean. We aimed to elucidate its role in soybean growth and development using existing gene-edited (knockout) and overexpression materials available in our laboratory. Preliminary analyses revealed that *GmSAUR46b* exhibits dynamic expression patterns in response to light–dark transitions, suggesting its potential involvement in light-dependent regulatory pathways. To systematically dissect the function and molecular mechanism of *GmSAUR46b*, we employed a multidimensional approach combining genetic manipulation, transcriptome profiling, and phenotypic characterization. RNA sequencing (RNA-Seq) was performed on CRISPR/Cas9-mediated knockout (KO) lines and 35S-driven overexpression (OE) lines of *GmSAUR46b*, alongside wild-type (WT) soybean (cv. Williams 82, W82), across three key tissues—shoot apical meristems (SAMs), stems, and leaves—at three developmental stages (V1, V2, V3). This approach allowed us to capture tissue- and stage-specific gene expression dynamics associated with *GmSAUR46b* perturbation. Transcriptomic analyses, including identification of differentially expressed genes (DEGs) and functional enrichment (GO, KEGG, KOG), were complemented by the discovery and characterization of novel transcripts, providing insights into both known and unannotated regulatory elements. Given that the thickness of the leaf midrib directly impacts the mechanical support and vascular transport, while stem trichomes serve as physical barriers against biotic and abiotic stresses [75,76], we further validated the transcriptome analysis results by conducting anatomical studies using paraffin sectioning and scanning electron microscopy (SEM) to assess the leaf midrib thickness and stem trichome density under varying light conditions. These analyses indicate that *GmSAUR46b* plays a significant regulatory role in shaping soybean morphology.

By integrating these datasets, we aimed to address the following three core objectives: (1) to define the role of *GmSAUR46b* in mediating light-responsive growth and development in soybean; (2) to identify the downstream molecular pathways—particularly those involved in hormone metabolism, cell wall modification, and photosynthesis—that are regulated by *GmSAUR46b*; and (3) to characterize the tissue-specific and light-dependent nature of these regulatory mechanisms. Ultimately, this research not only advances our understanding of the functional diversity of *SAUR* genes in soybean but also provides valuable insights for the genetic improvement and molecular breeding of soybean, with the potential to enhance the adaptability and yield under varying environmental conditions.

## 2. Results

### 2.1. Identification and Functional Screening of GmSAUR46b as a Candidate Gene Responsive to Light Signaling

**Figure 1:**
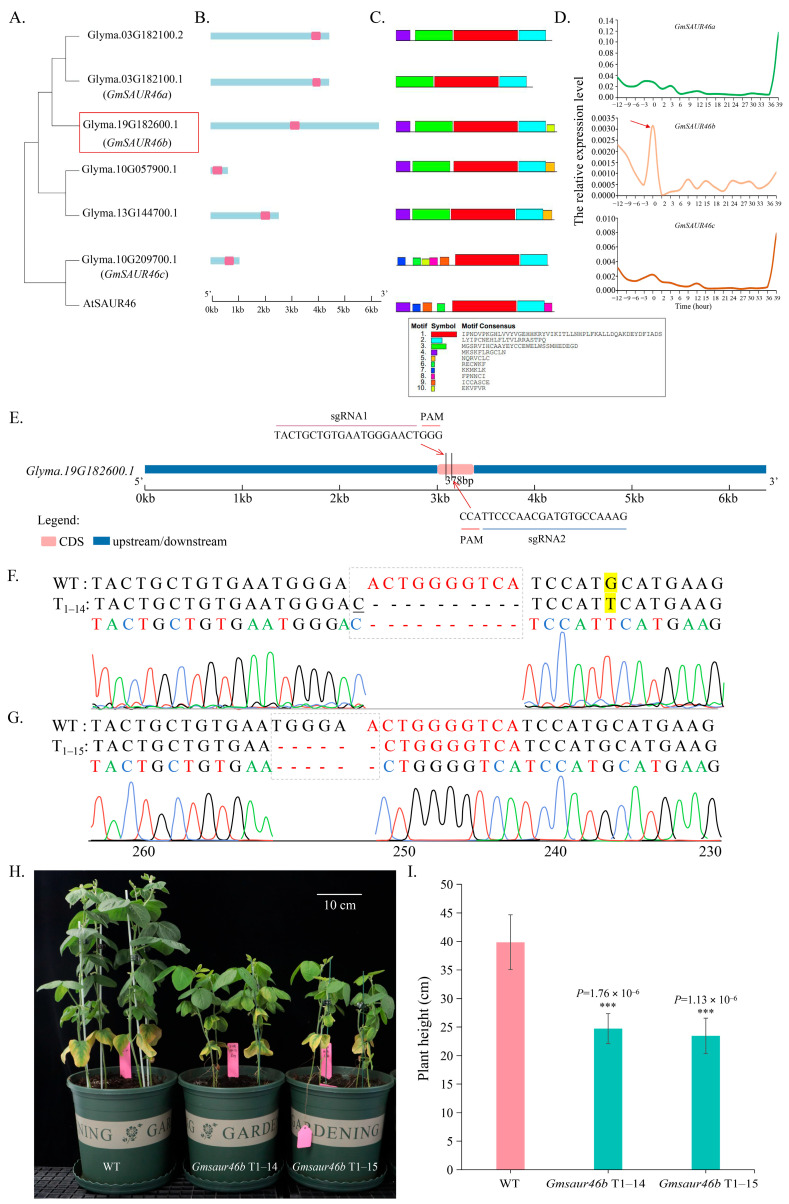
The response of *GmSAUR46b* to light signals and its regulation of plant height. (**A**–**C**) Evolutionary analysis (**A**), gene structure (**B**), and conserved domain analysis (**C**) of *GmSAUR46s* in soybean. (**D**) Analysis of the light-responsive expression pattern of *GmSAUR46s*. Data are presented as means ± SD of three replicates. The box and arrow represent the gene *GmSAUR46b*. (**E**) The sgRNA sequencing of the CRISPR-Cas9 of *GmSAUR46b*. (**F**,**G**) Identification of the gene-editing status of the T1 generation *GmSAUR46b* lines numbered 14 (**F**) and 15 (**G**) in soybeans. Different colored curves represent different bases. The shaded part is highlighted by a dashed line box, representing base deletions, and the yellow-marked bases indicate base mutations, and the underlined bases represent single-base insertions. (**H**–**I**) The regulation of soybean plant height by the *GmSAUR46b* gene-editing lines (numbered 14 and 15) of the T1 generation (**H**) and their statistical analysis (**I**). Scale bar, 10 cm. The data are presented as means ± SD of 8 samples of the same line. *** *p* < 0.001.

The genomic sequence alignment across the soybean genome identified six homologous genes of *GmSAUR46* (Figure 1A–C). qRT-PCR analysis showed that the expression of the gene with the ID *Glyma.19G182600.1* (designated as *GmSAUR46b*) sharply increased at the onset of light and dark treatment, then rapidly decreased thereafter. This gene was significantly responsive to light signals (Figure 1D), suggesting its role in light-dependent regulation.

Preliminary phenotypic characterization revealed that the *GmSAUR46b* knockout lines exhibited significantly reduced plant height compared to the wild type (Figure 1E–I). To investigate the role of *GmSAUR46b* in soybean growth and development, and to elucidate its underlying molecular mechanisms, we utilized the existing *GmSAUR46b* KO and OE lines available in our laboratory for functional validation. SAMs, leaves, and stems were collected from the *GmSAUR46b* KO and OE lines at three developmental stages (V1, V2, and V3) for transcriptome sequencing. Through transcriptome analysis, we aimed to clarify the function of *GmSAUR46b* in soybean growth and development and to identify its potential molecular mechanisms.

### 2.2. Identification and Characterization of Novel Transcripts

Using StringTie software (v2.2.1) for transcript assembly, new transcripts and genes were successfully identified. The types and quantities of these novel transcripts are summarized in Table 1. Among them, the “j” type (multi-exon transcripts with at least one matching exon) is the most abundant, totaling 33,394. Additionally, there are 4204 “o” types (transcripts partially overlapping with other reference exons on the same strand), 3353 “u” types (unknown novel transcripts), 1543 “x” types (exon overlaps on the opposite strand), and 260 “i” types (transcripts completely contained within the introns of the reference transcripts) (Table 1).

TransDecoder was used to predict the coding sequences of the newly identified transcripts, and the coding sequence (CDS) length distribution is presented in Figure S1. The statistical results of the functional annotation of the novel transcripts are shown in Table S1. A total of 42,754 transcript sequences were analyzed, of which 38,187 (89.32%) received at least one type of annotation; 27,677 (64.74%) were annotated in the KEGG database; 13,456 (31.47%) were annotated in the KEGG Pathway database; and 38,161 (89.26%) were annotated in the Nr database.

### 2.3. Functional Annotation of New Transcripts via GO, KEGG, and KOG Analyses

**Figure 2:**
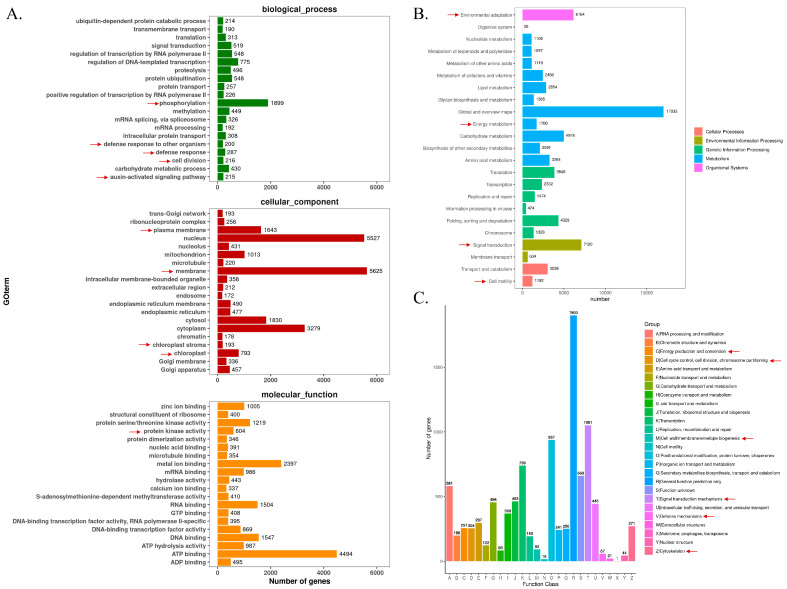
Gene Ontology (GO), Kyoto Encyclopedia of Genes and Genomes (KEGG), and euKaryotic Orthologous Groups (KOG) annotations for the new transcripts. (**A**) GO annotation secondary classification distribution chart. (**B**) KEGG annotation of transcript sequences. (**C**) Direct homology classification of transcripts using the KOG database. The red arrows represent the enriched pathways related to photosynthesis, hormone signaling, cell division, nuclear cell elongation.

To elucidate the functional roles of the new transcripts in key biological processes—including stress resistance, hormone metabolism, photosynthesis, chloroplast and thylakoid development, trichome density, and stem elongation—we performed Gene Ontology (GO), Kyoto Encyclopedia of Genes and Genomes (KEGG), and euKaryotic Orthologous Groups (KOG) annotations on the new transcripts. These annotation were subsequently classified according to the KEGG metabolic pathways (Figure 2).

For the GO annotation (Figure 2A), within the “biological process” category, terms such as “defense response”, “phosphorylation”, “cell division”, and “auxin-activated signaling pathway” were significantly enriched. The “defense response” is closely associated with stress resistance. “Protein phosphorylation” serves as a key mechanism in signal transduction and may participate in hormone-related signaling pathways. In the “cellular component” category, a substantial number of genes were assigned to both “plasma membrane” and “chloroplast”. The “plasma membrane” functions as a critical site for hormone perception and stress signal reception, while the “chloroplast” is the core organelle for photosynthesis, suggesting roles in chloroplast development and photosynthetic processes. At the “molecular function” level, “protein kinase activity” and “ATP binding” were prominent. “Protein kinase activity” is essential for mediating signal transduction cascades that regulate hormone metabolism, and “ATP binding” provides energy support for various biological processes, including photosynthesis and cell growth related to stem elongation and trichome formation.

Regarding the KEGG annotation (Figure 2B), the new transcripts were categorized into functional classes, including cellular processes, environmental information processing, genetic information processing, metabolism, and organismal systems. In terms of “stress resistance”, the “Environmental adaptation” pathway contained 1680 transcripts, suggesting a substantial role in enabling the organism to cope with environmental stresses. For “hormone metabolism and signal transduction”, the “Signal transduction” pathway (with 1744 transcripts) likely mediates hormone-related signaling, which is fundamental for regulating hormone levels and their downstream effects on growth and development. Concerning “photosynthesis, chloroplast development, and thylakoid development”, although no single pathway exclusively corresponds to all these processes, the “Energy metabolism” pathway (with 424 transcripts) is critical for providing energy. Additionally, pathways involved in cellular component biosynthesis may contribute to the construction of chloroplasts and thylakoids, as well as support photosynthetic reactions. For “trichome number and stem elongation”, which are associated with cell growth and development, the “Cell motility” pathway (with 392 transcripts), along with pathways related to cell cycle regulation, might influence trichome formation and stem elongation by modulating cell division, expansion, and movement. Collectively, the GO and KEGG annotations provide comprehensive insights into the potential involvement of transcripts in the targeted biological processes, laying a solid foundation for subsequent in-depth investigations.

For the KOG annotation (Figure 2C), the new transcripts were categorized into various functional groups, and the gene counts within each group were analyzed. For “stress resistance”, 57 genes were assigned to “Defense mechanisms” (category V), providing a foundation for exploring stress-responsive regulation. Regarding “hormone metabolism and signal transduction”, the “Signal transduction mechanisms” group (category T) contained 1051 genes, which may be involved in mediating hormone-related signaling pathways. Concerning “photosynthesis, chloroplast development, and thylakoid development”, although no single category directly corresponds to all these processes, genes in groups related to energy production (such as “Energy production and conversion”, category C) and cellular structure (such as “Cell wall/membrane/envelope biogenesis”, category M) could potentially participate in these chloroplast-associated events. For “trichome number and stem elongation”, which are related to cell growth and development, genes in “Cell cycle control, cell division, chromosome partitioning” (category D) and “Cytoskeleton” (category Z) might play roles in regulating these traits. This functional classification provides insights into the potential involvement of the new transcripts in the biological processes we focused on, guiding the subsequent targeted studies.

### 2.4. Evolutionary and Genomic Distribution Analyses of Transcripts

**Figure 3:**
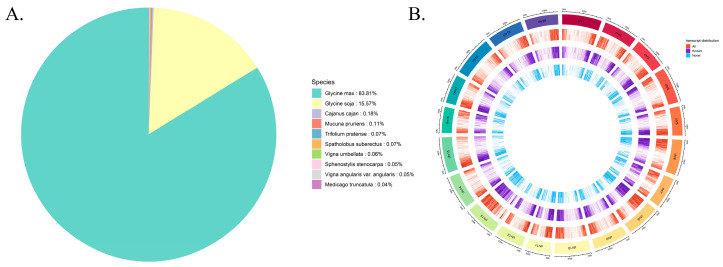
(**A**) Nr-annotated species distribution map. (**B**) Transcript density distribution map. From outside to inside, they are the chromosomes, all transcripts, known transcripts, and new transcripts, and the positive and negative strands are distinguished.

To further investigate the evolutionary relationships and genomic distribution patterns of the transcripts, we analyzed the species distribution based on the Nr annotation (Figure 3A) and transcript density across chromosomes (Figure 3B). For the Nr-annotated species distribution, the transcripts showed the highest similarity to sequences from “*Glycine max*” (83.81%), followed by “*Glycine soja*” (15.57%), with only a small proportion matching sequences from other species such as “*Cajanus cajan*” (0.18%), “*Mucuna pruriens*” (0.07%), and “*Trifolium pratense*” (0.07%). This high homology to “*Glycine max*” and “*Glycine soja*” indicates a close evolutionary relationship, providing a reliable reference for the subsequent functional inference based on known genes from these related species. Regarding the transcript density distribution map, from the outermost to the innermost layers, it depicts the chromosomes, all transcripts, known transcripts, and novel transcripts, while also distinguishing between positive and negative strands. The transcripts, including both known and novel ones, exhibit an uneven distribution across different chromosomes. Notably, regions with relatively high densities of all the transcripts were observed, and the distribution patterns of the known and novel transcripts showed both overlaps and differences, suggesting complex transcriptional regulation and potential novel transcriptional events in specific chromosomal regions. Collectively, the Nr annotation clarifies the evolutionary context of the transcripts, and the chromosome-level density distribution provides insights into their genomic organization, with the two analyses complementing each other to enhance our understanding of the transcripts.

### 2.5. Distribution of Gene Expression Levels Across Samples

**Figure 4:**
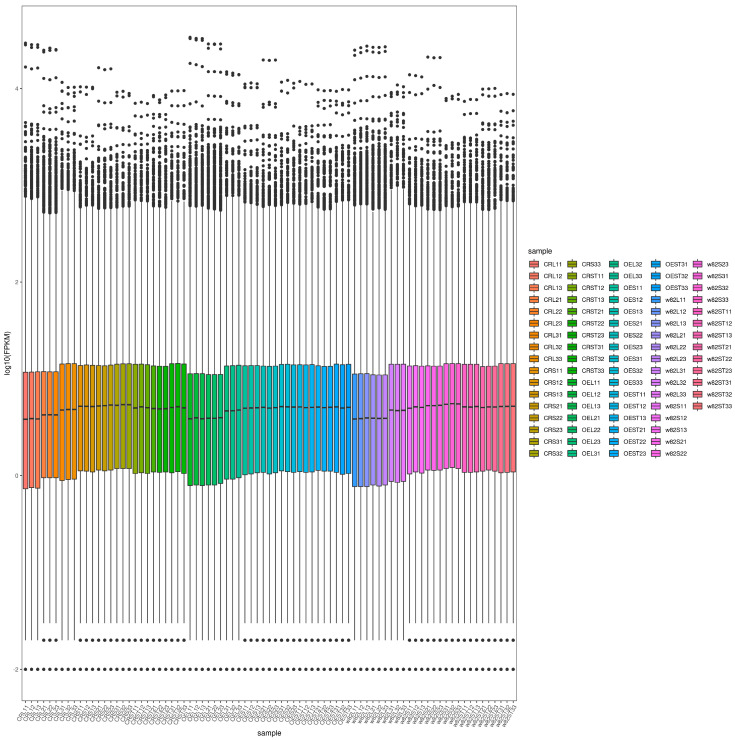
Box plot of gene expression. This composite plot illustrates the distribution of the gene expression levels across different samples. The upper portion shows individual gene expression data points, while the lower box-and-whisker plot displays key statistical summaries (maximum, upper quartile, median, lower quartile, and minimum) of the gene expression for each sample group. Different colors represent distinct sample categories, allowing for the comparison of the gene expression patterns and variability among various samples.

To visualize the distribution of the gene expression levels across samples, we generated the FPKM density distribution graph (Figure S2) and the box plot (Figure 4). The box plot illustrates the distribution of the gene expression levels across samples, with each box displaying five key statistics from top to bottom: maximum value, upper quartile, median, lower quartile, and minimum value. As shown, the median values across different samples were relatively consistent, indicating a stable overall trend in gene expression within the transcriptomic profiles. Additionally, the ranges between the maximum and minimum values, as well as the interquartile ranges, varied among the samples, reflecting differences in the dispersion of gene expression levels. These variations may be associated with the distinct genetic backgrounds of *GmSAUR46b* mutant materials.

### 2.6. Transcriptomic Analysis Reveals Enrichment of DEGs in Hormone and Photosynthesis Pathways

**Figure 5:**
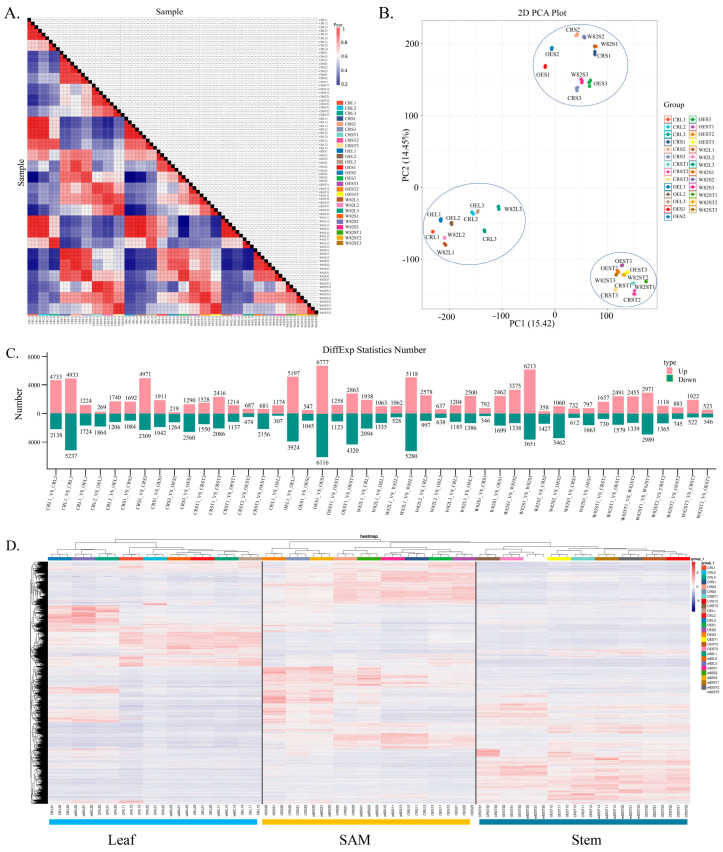
Analysis of differentially expressed genes (DEGs). (**A**) Analysis of sample correlation. (**B**) Principal component analysis (PCA). (**C**) Statistical analysis of DEGs. (**D**) Cluster analysis of DEGs. Heatmaps are drawn based on the expression levels of genes in each sample, with each column representing a sample and each row representing a gene in the map. W82, Williams 82; CR, the CRISPR-Cas9-edited lines of *GmSAUR46b*; OE, the 35S overexpression mutant lines of *GmSAUR46b*; L, leaf; S, shoot apical meristem (SAM); ST, stem; 1, the stage when the first trifoliate leaf of soybean unfolds and flattens; 2, the stage when the second trifoliate leaf of soybean unfolds and flattens; 3, the stage when the third trifoliate leaf of soybean unfolds and flattens; Up, up-expressed DEGs; Down, down-expressed DEGs.

To assess the transcriptomic variations among samples, we first performed clustering analysis (Figure 5A), which revealed distinct clustering patterns that reflected the relationships between samples. The 2D PCA plot (Figure 5B) further illustrated the sample distribution, with PC1 (15.42%) and PC2 (14.45%) capturing the major sources of variation. Samples within the same group tended to cluster together, indicating strong biological reproducibility. Subsequently, we analyzed the number of DEGs across various comparison groups (Figure 5C). The results showed that the number of DEGs varied significantly among the different comparison combinations. For all the DEGs, clustering analysis was conducted on both samples and genes based on their expression profiles, with the results presented as a heatmap (Figure 5D). In this heatmap, each column represents a sample, and each row represents a gene. Notably, these genes clustered into three distinct groups that corresponded well with the three tissue types: leaf, SAM, and stem. This clustering pattern indicates that the differentially expressed genes exhibit tissue-specific expression, suggesting that *GmSAUR46b* may regulate soybean growth and development through tissue-specific modulation of gene expression in leaves, SAMs, and stems.

### 2.7. Functional Enrichment Analysis of DEGs

**Figure 6:**
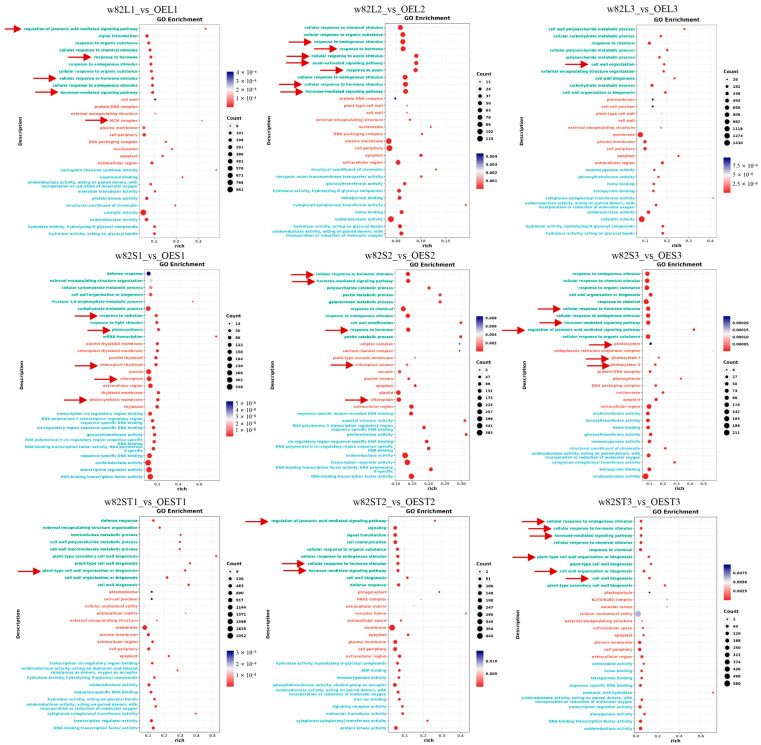
GO functional enrichment analysis of differentially expressed genes based on RNA-Seq sequencing results. W82, Williams 82; L, leaf; S, shoot apical meristem; ST, stem; 1, the stage when the first trifoliate leaf of soybean unfolds and flattens; 2, the stage when the second trifoliate leaf of soybean unfolds and flattens; 3, the stage when the third trifoliate leaf of soybean unfolds and flattens; the red arrow, the enriched pathway related to hormone metabolism; OE, overexpression lines.

To elucidate the functional roles of the DEGs, GO enrichment analysis was performed for each comparison group (Figure 6 and Figure S3). Notably, despite variations in the number of DEGs across the different comparison groups (Figure 5C), these DEGs were significantly enriched in several key biological processes. Specifically, they were predominantly associated with terms related to “cell wall formation and metabolism” (such as “cell wall organization”, “plant type cell wall organization or biogenesis”), “hormone metabolism and response” (including “regulation of jasmonic acid mediated signaling pathway”, “response to hormone”, “hormone-mediated signaling pathway”, “response to endogenous stimulus”, “response to auxin”), and “photosynthetic systems and chloroplast development” (like “photosynthesis”, “chloroplast”, “photosynthetic membrane”, “chloroplast stroma”, “photosystem”, “photosystem I”, “photosystem II”) (Figure 6 and Figure S3). Importantly, these findings are consistent with the previously described GO, KEGG, and KOG analyses of the transcripts (Figure 2), collectively indicating that cell wall-related processes, hormone-associated pathways, and photosynthetic machinery play critical roles in the observed transcriptomic changes.

### 2.8. Analysis of Alternative Splicing Events and Transcription Factor Families

To investigate the alternative splicing (AS) events and transcription factor (TF) families, we first analyzed the distribution of AS event types. Statistical plots were generated for different AS types in each sample (Figure S4A), identifying multiple AS event types, including A3SS, A5SS, MXE, RI, and SE, each exhibiting distinct proportions across samples. We then used rMATS to detect differential AS events among samples and counted the number of differential AS events for the comparison groups (Figure S4B). The distribution patterns of the AS event types differed from the overall sample distribution, indicating that AS events may be regulated in a group-specific manner. Additionally, the TF families were analyzed by performing TF prediction and identification using the PlantTFDB (Figure S4C). Various TF families were detected, with C3H accounting for 12.58%, MYB_related for 11.99%, MYB for 9.70%, and other families such as HB—other (6.62%) and HD-ZIP (5.96%) also representing notable proportions. These results suggest the diversity and complexity of the TF families involved in regulatory processes.

### 2.9. GmSAUR46b Specifically Regulates the Leaf Midrib Thickness in a Light-Dependent Manner and Modulates the Epidermal/Mesophyll Responses to Light Signals

To further validate the role of *GmSAUR46b* in soybean growth and development, we subjected the *GmSAUR46b* KO and OE lines, along with WT plants, to different light conditions (Figure S5). This approach was based on prior functional enrichment and expression pattern analyses of transcriptome data, aiming to assess their responses to light signals. Following treatment under varying light regimes, significant changes in leaf development were observed. Therefore, paraffin sectioning was employed to investigate the impact of *GmSAUR46b* on the anatomical structure of soybean leaves.

**Figure 7:**
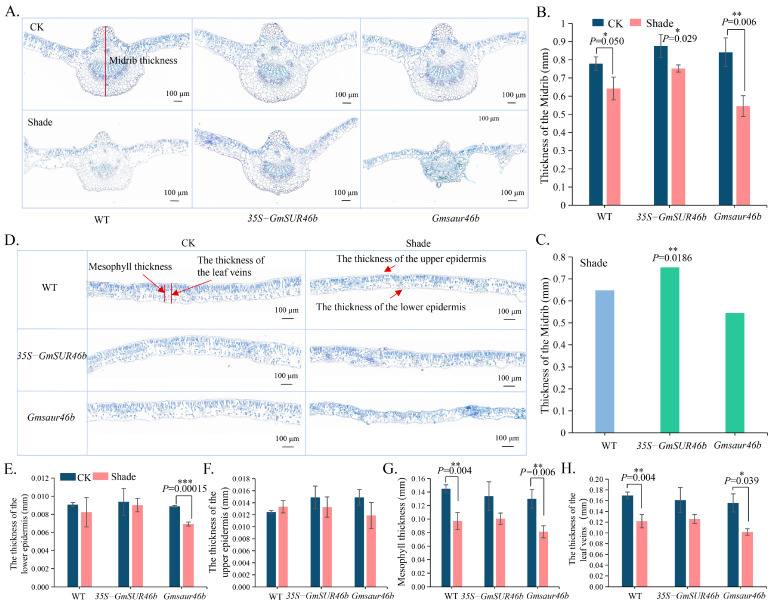
*GmSAUR46b* regulates the soybean leaf anatomical structure in a light-dependent manner. (**A**) Paraffin sections of leaf midribs from wild-type (WT), 35S-*GmSAUR46b* overexpression (OE), and *Gmsaur46b* knockout (KO) plants under control (CK, normal light) and shade conditions. Scale bar = 100 μm. (**B**) Statistical analysis of leaf midrib thickness under CK and shade conditions for WT, OE, and KO lines. (**C**) Statistical analysis of leaf midrib thickness under shade conditions for WT, OE, and KO lines. (**D**) Paraffin sections of leaf blades (showing mesophyll, upper epidermis, and lower epidermis) from WT, OE, and KO plants under CK and shade conditions. Scale bar = 100 μm. (**E**–**H**) Statistical analyses of lower epidermis thickness (**E**), upper epidermis thickness (**F**), mesophyll thickness (**G**), and leaf vein thickness (**H**) under CK and shade conditions for WT, OE, and KO lines. Data are presented as mean ± SD. *** *p* < 0.001, ** 0.001 ≤ *p* < 0.01, * 0.01 ≤ *p* < 0.05 (Student’s *t*-test).

The results showed that *GmSAUR46b* specifically regulates the central region of the leaf midrib (Figure 7, Table S2). Under normal light conditions, the thickness of the midrib in the *GmSAUR46b* OE lines was 0.88 mm, which is 11.36% greater than that in the WT plants (0.78 mm), although this difference was not statistically significant (*p* = 0.081). Similarly, the midrib central thickness in the *GmSAUR46b* KO lines was 0.84 mm, representing a 7.69% increase compared to the WT; however, no significant difference was observed (*p* = 0.284) (Figure 7A,B).

Shading treatment further enhanced the regulatory effect of *GmSAUR46b* on the midrib thickness, suggesting a light-dependent regulatory mechanism. Under shaded conditions, the midrib central thickness of the OE lines reached 0.75 mm, representing a significant increase of 15.38% compared to the WT plants (0.65 mm) under the same shading conditions (*p* = 0.019) (Figure 7C). Additionally, the midrib central thickness of the OE lines under shading was significantly reduced by 14.33% compared to that under normal light (*p* = 0.029) (Figure 7B). For the KO lines under shading, the midrib central thickness measured 0.545 mm, showing no significant difference from the WT under shading; however, it was significantly reduced by 35.71% compared to the KO lines under normal light (*p* = 0.0063) (Figure 7A–C). Collectively, these results indicate that *GmSAUR46b* plays a specific role in regulating the central thickness of the leaf midrib, and this regulation is strongly influenced by the light conditions.

Additionally, in the *GmSAUR46b* OE lines, there were no significant differences in the thickness of the upper epidermis, lower epidermis, mesophyll, or leaf blade between normal light and shade conditions. In contrast, the *GmSAUR46b* KO lines exhibited a significantly thinner lower epidermis, mesophyll, and leaf blade under shade conditions compared to normal light conditions. However, under both normal light and shade conditions, the thicknesses of these leaf structures in the KO lines did not differ significantly from those in the WT plants (Figure 7D–H). These results further support the specificity and light-dependent characteristics of *GmSAUR46b* in regulating the leaf structure. They also suggest that its function may be localized to specific structures such as the midrib, while its influence on the epidermis and mesophyll may require the combined action of other genes or environmental signals.

### 2.10. GmSAUR46b Regulates the Stem Trichome Density in a Light-Dependent Manner

**Figure 8:**
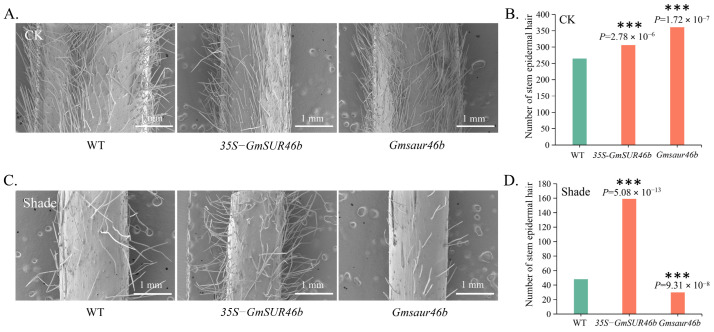
*GmSAUR46b* regulates the soybean stem epidermal hair density in a light-dependent manner. (**A**) Scanning electron microscopy (SEM) images of stem epidermal hairs from wild-type (WT), 35S-*GmSAUR46b* overexpression (OE), and *Gmsaur46b* knockout (KO) plants under control (CK, normal light) conditions. Scale bar, 1.00 mm. (**B**) Statistical analysis of stem epidermal hair number per unit area under CK conditions for WT, OE, and KO lines. (**C**) SEM images of stem epidermal hairs from WT, OE, and KO plants under shade conditions. Scale bar, 1.00 mm. (**D**) Statistical analysis of stem epidermal hair number per unit area under shade conditions for WT, OE, and KO lines. Data are presented as mean ± SD. *** *p* < 0.001 (Student’s *t*-test).

SEM analysis was conducted to investigate the effect of *GmSAUR46b* on the stem trichome density under normal light (CK) and shade conditions. Under CK conditions, both the *GmSAUR46b* OE and KO lines exhibited significantly increased stem trichome density compared to the WT plants (Figure 8A,B, Table S3). The average number of trichomes per square millimeter of stem surface in the OE lines was 306, representing a highly significant increase of 13.40% compared to the WT (265 trichomes per square millimeter, *p* = 2.78 × 10^−6^). The KO lines had an average of 361 trichomes per square millimeter of stem surface, showing a highly significant increase of 36.23% compared to the WT (*p* = 1.72 × 10^−7^).

However, under shaded conditions, the regulatory effect of *GmSAUR46b* on the stem trichome density changed. The OE lines maintained a relatively high trichome density, averaging 159 trichomes per square millimeter of stem surface, representing a highly significant increase of 231.25% compared to the WT, which had 48 trichomes per square millimeter (*p* = 5.08 × 10^−13^). In contrast, the KO lines averaged 30 trichomes per square millimeter, showing a high significant reduction of 37.50% compared to the WT (*p* = 9.31 × 10^−8^) (Figure 8C,D). These results indicate that *GmSAUR46b* plays a crucial role in regulating the stem trichome density, and this regulation is strongly influenced by the light conditions.

### 2.11. Differentially Expressed Genes Associated with Observed Morphological Phenotypes

To further elucidate the molecular basis of the observed phenotypic changes (dwarfism, altered leaf midrib thickness, and stem trichome density), we integrated RNA-seq data with phenotypic traits to identify key downstream genes potentially mediating these phenotypes.

**Figure 9:**
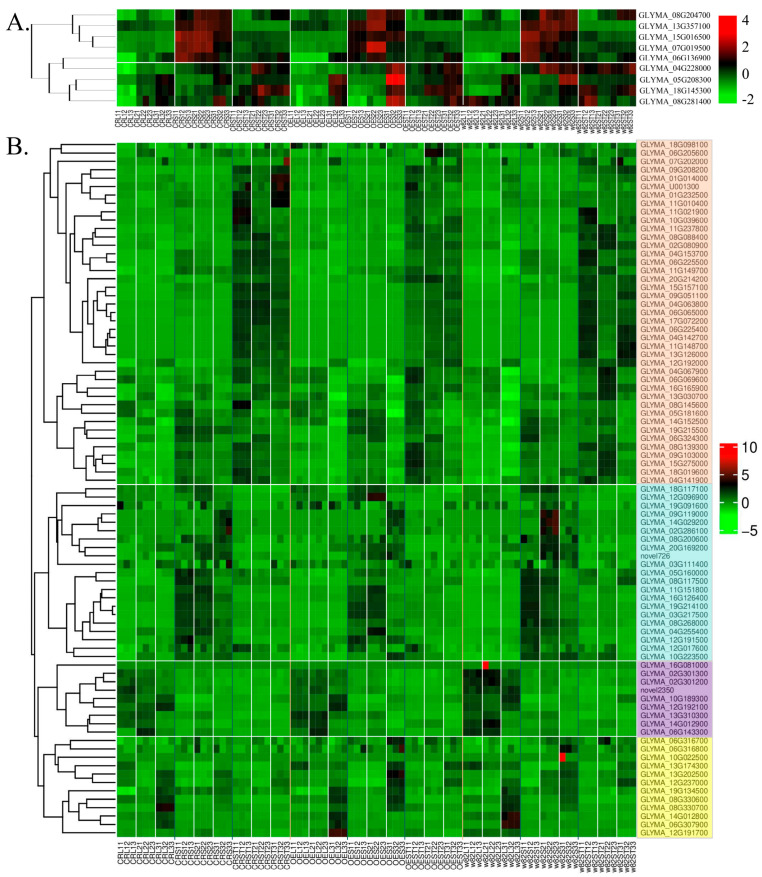
Expression patterns of genes related to trichome development and cell wall modification. (**A**) Expression of *GLABRA* homologs (including *GLYMA_08G204700*, *GLYMA_13G357100*, etc.) in different tissues and genotypes at the V1–V3 stages. (**B**) Expression of cellulose synthase genes classified into four classes, with the relative expression levels shown.

For the stem trichome density, a trait associated with trichome development, several *GLABRA* homologs exhibited distinct expression patterns across genotypes (*GmSAUR46b* gene-edited, overexpression, and wild-type) and tissues (leaves, SAMs, and stem) at developmental stages V1, V2, and V3 (Figure 9A). Notably, *GLYMA_08G204700*, *GLYMA_13G357100*, *GLYMA_15G016500*, *GLYMA_07G019500*, and *GLYMA_06G136900* showed significantly higher expression in the SAM compared to the stems and leaves, with the highest levels observed in the overexpression lines at stage V2 (Figure 9A). Given the established role of *GLABRA* genes in trichome initiation and development, these upregulated homologs likely contribute to the increased stem trichome density observed in the overexpression lines.

Regarding the cell-wall-related phenotypes, such as dwarfism and altered leaf midrib thickness, numerous cellulose synthase genes displayed differential expression profiles (Figure 9B). Based on their preferential expression in specific tissues and developmental stages, these genes were categorized into four classes: Class I (stem-preferential), Class II (SAM-preferential), Class III (leaf-preferential), and Class IV (SAM-specific at stage V3) (Figure 9B). These tissue- and stage-specific expression patterns suggest that these cellulose synthase genes may regulate cellulose synthesis, with disruptions to their expression potentially impairing stem elongation (leading to dwarfism) and leaf midrib structural integrity.

Collectively, these differentially expressed genes—*GLABRA* homologs (involved in trichome development) and cellulose synthases (associated with cell wall modification)—are strong candidates linking genetic perturbations to the observed morphological traits.

## 3. Discussion

### 3.1. The Role of GmSAUR46b in Light-Responsive Soybean Development

Light is a critical environmental cue that regulates numerous aspects of plant growth and development [3,4,5,6,7,14,15,16,17,18]. Our study identified *GmSAUR46b* as a key regulator involved in light-dependent soybean development. The dynamic expression pattern of *GmSAUR46b* in response to light–dark transitions (Figure 1D) suggests its involvement in light signaling pathways. Phenotypic analysis further revealed that the *GmSAUR46b* knockout lines exhibited extreme dwarfism (Figure 1E–I), underscoring its essential role in regulating plant height, consistent with previous findings on *SAUR* genes mediating plant height [38,39].

Transcriptomic profiling of the *GmSAUR46b*-modified lines across multiple tissues and developmental stages revealed extensive alterations in gene expression related to hormone metabolism, cell wall modification, and photosynthesis (Figure 6 and Figure S3). GO enrichment analysis identified significant overrepresentation of DEGs in terms associated with “cell wall organization”, “hormone-mediated signaling pathways”, and “photosynthetic systems” (Figure 6 and Figure S3). These findings align with the well-established functions of *SAUR* genes in cell elongation [27,28,29,30,32,34,35,36], hormone signal transduction [46,47,48,49], and light response [50,51]. For instance, SAUR proteins have been implicated in regulating H^+^-ATPases and K^+^ channels to control cell expansion and stomatal movements [27,56], processes closely linked to cell wall dynamics and photosynthesis. Therefore, *GmSAUR46b* likely integrates light signals with hormone and cell wall metabolic pathways to modulate soybean growth.

### 3.2. Specific Regulation of Leaf Midrib Thickness by GmSAUR46b

The leaf midrib thickness is crucial for mechanical support and vascular transport, directly influencing the leaf function and overall plant performance. Our anatomical analysis revealed that *GmSAUR46b* specifically regulates the central thickness of the leaf midrib in a light-dependent manner (Figure 7). Under normal light conditions, the *GmSAUR46b* OE lines exhibited a slight increase in midrib thickness, while the KO lines showed only marginal effects, with no significant differences compared to the WT. However, under shade conditions, the OE lines demonstrated a significant increase in midrib thickness relative to the WT, whereas the KO lines displayed a pronounced reduction compared to their counterparts under normal light (Figure 7A–C).

This light-dependent regulation suggests that *GmSAUR46b* fine-tunes midrib development in response to light availability. Under shade conditions, where light is limited, the increased midrib thickness observed in the OE lines may enhance vascular efficiency or mechanical support, compensating for the reduced photosynthetic capacity. Conversely, the thinner midrib in the shaded KO lines could impair these functions, potentially contributing to the observed dwarf phenotype. The specificity of *GmSAUR46b* in regulating the midrib, with no significant effects on epidermal or mesophyll thickness in the OE lines (Figure 7D–H), indicates a targeted role in midrib development, possibly by modulating cell division or expansion in midrib vascular tissues.

### 3.3. Light-Dependent Regulation of Stem Trichome Density

Stem trichomes serve as physical barriers against biotic and abiotic stresses [75,76,77], and their development is frequently influenced by environmental cues. Our SEM analysis revealed that *GmSAUR46b* regulates the stem trichome density in a light-dependent manner (Figure 8). Under normal light conditions, both the OE and KO lines exhibited significantly higher trichome densities than the WT (Figure 8A,B), suggesting that *GmSAUR46b* is part of a complex regulatory network controlling trichome initiation, where either its overexpression or knockout disrupts the balance.

**Figure 10:**
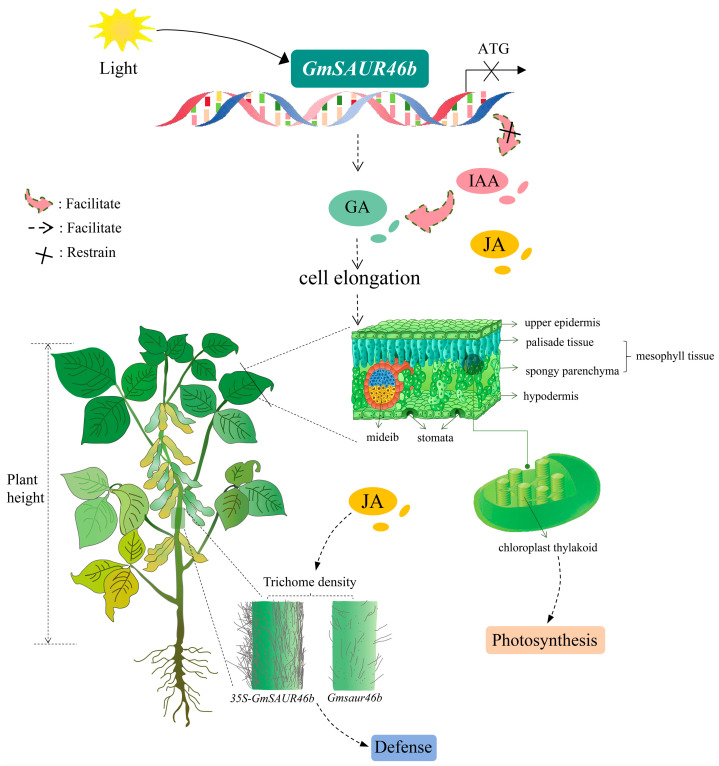
A proposed model illustrating *GmSAUR46b*-mediated regulation of soybean growth, development, and stress responses. Light signals activate *GmSAUR46b*, which integrates gibberellin (GA) [7], auxin (IAA), and jasmonic acid (JA) signaling pathways to modulate cell elongation. This regulatory cascade influences multiple agronomic and physiological traits: plant height, leaf anatomical features (including midrib structure, mesophyll tissue, and chloroplast thylakoid development), photosynthesis, stem trichome density (with 35S-*GmSAUR46b* overexpression and *GmSAUR46b* knockout lines as exemplars), and defense responses. Arrows indicate facilitation, dashed lines indicate regulatory links, and crosses indicate restraint. ATG, a translation initiation factor involved in related regulatory processes.

Under shade conditions, the regulatory pattern shifted: the OE lines maintained high trichome density, whereas the KO lines exhibited a drastic reduction (Figure 8C,D). This suggests that *GmSAUR46b* promotes trichome development under shade, possibly as an adaptive response to enhance stress resistance in low-light environments. The contrasting effects of *GmSAUR46b* overexpression and knockout under shade highlight its central role in integrating light signals to modulate trichome density, potentially involving crosstalk with hormone pathways. Both auxin and jasmonic acid (JA) are known regulators of trichome development [77,78] and play multifaceted roles in plant abiotic stress responses [79,80,81,82,83,84]. For instance, auxin and JA can induce the formation of type II, V, and VI trichomes in tomato [77]. Under mitochondrial translational stress, JA mediates retrograde signaling to balance plant growth and defense responses [85]. JA priming enhances the antioxidant defense system and photosynthetic capacity in soybean, alleviating the adverse effects of combined heat and drought stress [86]. Additionally, in rice, JA participates in phosphorus remobilization in root cell walls via a nitric-oxide-dependent pathway [87]. JA, salicylic acid, and GA interact to induce trichome development in *A. thaliana* [88]. Furthermore, the results of the GO and KEGG functional enrichment analyses in this study revealed that the DEGs were significantly enriched in pathways such as “response to auxin and jasmonic acid”, “response to endogenous stimulus”, “hormone response”, and “light signaling, photosynthesis, chloroplast thylakoid membrane development”. This further indicates that in soybean, *GmSAUR46b* may integrate light signals and hormone, especially JA and auxin, to regulate trichome development, thereby contributing to the stress resistance regulation of stress resistance (Figure 10).

### 3.4. Novel Transcripts and Functional Annotation

Our transcriptome analysis identified a substantial number of novel transcripts (Table 1, Figure S1), with significant annotations in the KEGG, KOG, and Nr databases (Table S1). Enrichment analyses of these transcripts and DEGs using GO, KEGG, and KOG consistently highlighted pathways related to stress resistance, hormone metabolism, and photosynthesis (Figure 2). The high sequence homology of the transcripts to *Glycine max* and *Glycine soja* (Figure 3A) underscores their evolutionary conservation, while the uneven chromosomal distribution (Figure 3B) suggests spatial regulation of transcriptional activity.

These novel transcripts expand the repertoire of known soybean genes and offer candidates for further investigation of *GmSAUR46b*-mediated regulation. For example, novel transcripts associated with “defense response” or “hormone signaling” may act downstream of *GmSAUR46b* to modulate stress tolerance or growth. The identification of these transcripts deepens our understanding of the molecular complexity underlying *GmSAUR46b* function.

In summary, our findings suggest that *GmSAUR46b* functions as a central hub integrating light signals with hormone metabolism, cell wall modification, and stress response pathways to regulate soybean growth and development (Figure 10). Under varying light conditions, *GmSAUR46b* modulates specific traits such as the leaf midrib thickness and stem trichome density, likely through tissue-specific regulation of gene expression. The specificity of *GmSAUR46b* in regulating midrib and trichome development, coupled with its influence on broader pathways such as hormone signaling and photosynthesis, highlights the multifaceted nature of *SAUR* gene function. Understanding *GmSAUR46b*’s role in light-dependent development has practical implications for soybean breeding. Modulating *GmSAUR46b* expression could potentially enhance soybean adaptability to variable light conditions (e.g., shade tolerance in dense canopies or resistance to high light stress). Our study provides comprehensive insights into *GmSAUR46b*’s function in soybean, emphasizing its role in integrating light signals to regulate tissue-specific development and broader physiological pathways. These findings advance our understanding of *SAUR* gene biology in soybean and offer promising avenues for molecular breeding.

## 4. Materials and Methods

### 4.1. Construction of Gene Knockout Vector and Soybean Transformation

#### 4.1.1. CRISPR-Cas9 Vector Construction for *GmSAUR46b* Knockout

Gene editing was performed using the CRISPR-Cas9 system, referencing the soybean transformation protocol from Boyuan Bio (Wuhan, China). Specific single-guide RNAs (sgRNAs) targeting *GmSAUR46b* were designed (sequences shown in Figure 1E) and cloned into the BsaI restriction site of the PEG401 vector to construct the CRISPR-Cas9 expression vector. This vector was then introduced into the *Agrobacterium tumefaciens* strain EHA105 via electroporation.

Primer synthesis: Primers used to amplify the target sgRNA cassette were synthesized as follows:

Forward: cagtGGTCTCatgcatactgctgtgaatgggaactgttttagagc

Reverse: cagtGGTCTCaaaacctttggcacatcgttgggaatgcac

Restriction digestion and ligation: The ligation reaction (20 μL total volume) contained 8 μL of nuclease-free water, 2 μL of 10× buffer, 1 μL of BsaI/Eco31I, 1 μL of T4 DNA ligase, 4 μL of PEG401 vector, and 4 μL of purified PCR product. The reaction was incubated at 37 °C for 20 min, followed by 5 cycles of 37 °C for 10 min, and 20 °C for 10 min, then 37 °C for 20 min, and heat inactivation at 80 °C for 5 min.

Transformation and verification in *E. coli*: 5–10 μL of the ligation product were transformed into competent *E. coli* cells, which were then plated on LB agar containing kanamycin and incubated at 37 °C for 12 h. Twelve colonies were selected for colony PCR using PEG401-specific primers (forward: GCAACGCTCTGTCATCGTTACAAT; reverse: gcgattaagttgggtaacgccaggg). Colonies exhibiting the expected 7843 bp band were confirmed by sequencing.

Electroporation into *Agrobacterium*: 1 μL of recombinant plasmid was mixed with 50 μL competent *A. tumefaciens* EHA105 cells and transferred to an electroporation cuvette for electroporation. Afterward, 1 mL of LB broth was added, and the mixture was incubated at 30 °C with shaking (at 180 rpm) for 30 min. Subsequently, 50 μL of the culture were plated on LB agar and incubated in the dark at 30 °C for 48 h.

#### 4.1.2. *Agrobacterium*-Mediated Soybean Transformation for Knockout Lines

Soybean transformation was performed in six stages: seed germination, co-cultivation, induction of differentiation, redifferentiation, elongation, and rooting, with medium formulations [89,90,91].

Explant preparation: Soybean seeds were surface-sterilized and germinated under sterile conditions. Cotyledon node explants were harvested after 3–5 days.

*Agrobacterium* infection: Explants were immersed in an *A. tumefaciens* EHA105 suspension (OD600 = 0.5) for 2–3 min with gentle stirring, then transferred to filter paper-lined co-cultivation plates and incubated at 25 °C in the dark for 3–5 days.

Callus induction and differentiation: Healthy explants (with hypocotyl ends removed) were cultured on dedifferentiation medium under a 16 h light/8 h photoperiod for 7–10 days, with subculturing onto fresh medium. Embryoids were then transferred to redifferentiation medium and cultured for 21 days under the same photoperiod, with subculturing.

Shoot elongation and rooting: Embryoids were transferred to elongation medium and cultured for 21 days. When the shoots reached approximately 5 cm in length, they were transferred to rooting medium and cultured for an additional 21 days under the same photoperiod to promote root and stem meristem development.

Verification of transgenic plants: T0 plants were screened for Bar/pat protein using Bar test strips. Positive seedlings were acclimatized in soil at 27 °C under a 16 h light/8 h dark photoperiod for 3–4 weeks. T1 seeds were sown in soil at 26 °C and 60% humidity, maintained under a 16 h light/8 h dark photoperiod. Mutations and the presence of Cas9 were verified in T1 plants using the Hi-TOM platform with specific primers (Table S5), and the genotypes were confirmed by Hi-TOM sequencing (Figure 1F,G). We identified a gene-edited line with a homozygous 8 bp deletion and a single base mutation (T1-14), as well as a gene-edited line with a 6 bp deletion (T1-15) (Figure 1G).

#### 4.1.3. Construction of Overexpression Vector and Soybean Transformation

The CDS of *GmSAUR46b* was amplified by PCR (primers in Table S6) and cloned into the XbaI and SacI restriction sites of the PTF101-35S vector. The recombinant vector was transformed into *A. tumefaciens* EHA105 via electroporation. *Agrobacterium*-mediated transformation of soybean explants, subsequent culture (including callus induction, differentiation, elongation, and rooting), and acclimatization of positive seedlings were performed using the same protocol as described for the gene knockout lines.

### 4.2. Plant Materials and Growth Conditions

Soybean W82, *GmSAUR46b* overexpression lines (OE-1, OE-2), and CRISPR/Cas9-mediated knockout mutants (KO-1, KO-2) were used in this study. The OE lines were generated by transforming the CDS of the *GmSAUR46b* gene under the control of the 35S promoter. The KO lines were created by targeting the first exon of *GmSAUR46b*, resulting in frameshift mutations (Table S4).

OE lines were generated by transforming the CDS of *GmSAUR46b* gene under the control of the Cauliflower Mosaic Virus 35S promoter.

Growth conditions: Seeds were sown in an illuminated incubator with an 8 h light/16 h dark cycle at 26 °C and 60% relative humidity (Figure S5).

**Figure 11:**
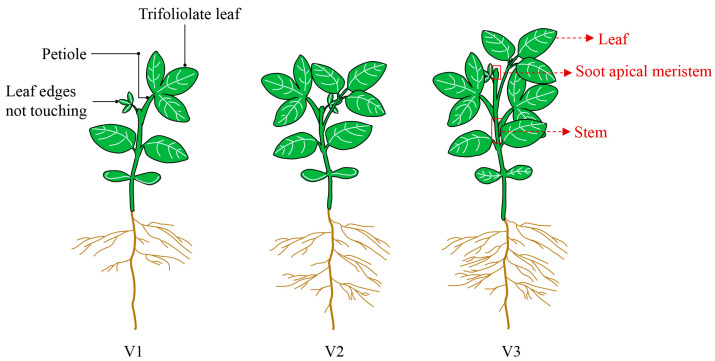
Schematic diagram of transcriptome sampling period. Leaves at three developmental stages: first trifoliate leaf unfolded (V1), second trifoliate leaf unfolded (V2), and third trifoliate leaf unfolded (V3).

For paraffin sectioning and Scanning Electron Microscopy (SEM) analyses, soybean plants (WT, OE and KO lines) were grown under two light regimes. Normal light: photosynthetic photon flux density (PPFD) = 314.5 μmol/(m^2^·s); red (600–700 nm) = 192.2 μmol/(m^2^·s); blue (400–500 nm) = 46.19 μmol/(m^2^·s); far-red (700–780 nm) = 53.07 μmol/(m^2^·s); R:B = 4.16. Simulated shading: PPFD = 149.8 μmol/(m^2^·s); red = 77.42 μmol/(m^2^·s); blue = 23.50 μmol/(m^2^·s); far-red = 170.4 μmol/(m^2^·s); R:B = 3.30. Growth conditions: 26 °C, 60% humidity, 8 h light/16 h dark. Samples were collected at the V3 stage (Figure 11).

### 4.3. Analysis of Gene Evolution, Structure and Conserved Motifs

The CDS and protein sequences of GmSAUR46s were obtained from the SoyBase database (http://www.soybase.org, accessed on 22 January 2025). Sequence alignment was performed using ClustalX (v1.8) [92,93]. Molecular evolutionary genetics analyses were conducted using the maximum likelihood, evolutionary distance, and maximum parsimony methods with MEGA5 software [94]. The gene structure was visualized using the GSDS 2.0 server [95]. MEME tools were employed to discover and analyze motifs [96,97].

### 4.4. Analysis of the Expression Patterns

As described in previous studies [98], the total RNA was extracted from fresh plant samples (minimum 2 g) that were immediately frozen in liquid nitrogen and pulverized into a fine powder. The extraction process was carried out with FastPure Universal Plant Total RNA Isolation Kit (Vazyme, Nanjing, China) [98], and 1 μg of the isolated total RNA was reverse-transcribed into first-strand cDNA using a HiScript II 1st Strand cDNA Synthesis Kit (Vazyme, Nanjing, China). qPCR-PCR reactions were carried out on a QuantStudio 5 Real-Time PCR System (Thermo Fisher Scientific, Waltham, MA, USA) with the ChamQ Universal SYBR qPCR Master Mix (Vazyme, Nanjing, China). Each 10 μL reaction system contained 5 μL SYBR Master Mix, 0.2 μL each of the forward and reverse primer, and 4.6 μL cDNA template. The thermal cycling parameters were configured as follows: an initial denaturation step at 95 °C for 30 s, followed by 40 cycles consisting of 95 °C denaturation for 10 s and 60 °C annealing/extension for 30 s [98]. The *GmTublin* gene (*Glyma.05G157300*) was used as the internal control [9], with the relative gene expression levels determined via the 2^−ΔΔCt^ algorithm. All the experiments included three biological replicates, each with three technical replicates. Primer sequences are provided in Supplementary Table S5.

### 4.5. RNA-Seq Sample Collection

For the transcriptome sequencing analyses of the leaf, stem and SAMs, seedlings were grown under SD conditions (8 h light/16 h dark) at 25 °C. Then, the leaf, stem, and SAMs (1.5 mm in length) were placed into liquid nitrogen for rapid freezing. For each tissue/stage, 8–10 biological replicates were collected from the WT, OE, and KO lines (total 81 samples: 3 genotypes × 3 tissues × 3 stages × 3 replicates) (Figure 11).

### 4.6. Transcriptome Data Analysis

Raw data processing: Raw reads were filtered using fastp (v0.21.0) [99] to remove adapters and low-quality reads, generating clean data. Quality control was verified using Fastqc (v0.11.9; Parameters: default). The clean reads were mapped to the Williams 82 reference genome [100,101] by HISAT2 (v2.2.1) [102] with the default settings for the parameters.

Alignment and expression quantification: Gene expression levels (Fragments Per Kilobase of transcript per Million mapped fragments, FPKM) were calculated using the feature Counts.

DEG identification: Differentially expressed genes (DEGs) between OE/WT and KO/WT were recognized using DESeq2 (Wolfgang Huber from Heidelberg University in Heidelberg, Germany) with the following thresholds: |log_2_(fold change)| > 1 and adjusted *p*-value (FDR) < 0.05 [103].

Functional enrichment: GO and KEGG enrichment analyses of the DEGs were carried out using clusterProfiler (Shanghai Institute of Biological Sciences, Chinese Academy of Sciences) [104,105], with the results visualized as bar plots and bubble plots. GSEA (v4.4.0) was used to identify enriched pathways.

### 4.7. Analysis of Novel Genes and Novel Transcripts

To systematically identify potential novel genes and transcripts in the samples, this study employed StringTie software (v2.2.1) [106] for de novo transcript assembly. The workflow was as follows: alignment results (in BAM format) of quality-controlled and alignment-filtered clean reads (refer to Section 4.6) mapped to the reference genome (version: Glycine_max_Glycine_max_v2.1_Ensemble_60_index) were used as input data. The transcript assembly parameters were set to the default, with a minimum transcript length of 200 bp and a minimum exon length of 50 bp to ensure the biological validity of the assembled products.

### 4.8. Observation and Measurement of Leaf Tissue Thickness

Referring to previous technical methods, samples were fixed in FAA fixative and then processed into qualified paraffin sections [98]. The sections were scanned using a PANNORAMIC whole-slide scanner (3DHISTECH, Budapest, Hungary) to generate complete image datasets, which were viewed with CaseViewer 2.4 (3DHISTECH, Budapest, Hungary). Target regions were imaged at 100× magnification under consistent background lighting. Using Image-Pro Plus 6.0 (Media Cybernetics, Rockville, MD, USA) with millimeters as the unit, five measurements per section were taken for the upper epidermis thickness, lower epidermis thickness, leaf vein thickness, mesophyll thickness (Table S2), and midvein center thickness at 100× magnification.

### 4.9. SEM Preparation and Analysis Procedures

For the SEM analysis, fresh tissues (≤3 mm^2^) were harvested within 1–3 min to minimize mechanical damage, rinsed with PBS (Servicebio, Wuhan, China, G0002) to remove contaminants, and labeled for target observation. Adherent cells on coverslips were rinsed with PBS after discarding the medium. Both sample types were fixed in Servicebio fixative (G1102) at room temperature for 2 h and stored at 4 °C. Post-fixation was performed using 1% osmium tetroxide (Ted Pella Inc., 18456, Redding, CA, USA) in 0.1 M PB (pH 7.4) for 1–2 h, followed by three 15 min rinses with 0.1 M PB. Dehydration was carried out using graded ethanol series (30–100%) and isoamyl acetate, each for 15 min, then the samples were dried with a Quorum K850 (Quorum Technologies, located in East Sussex, UK) critical point dryer. Finally, samples were sputter-coated with gold for approximately 30 s using a HITACHI MC1000 (Hitachi, Japan) and observed with a HITACHI SU8100 SEM (Hitachi, Japan), and take pictures at 30× magnification.

## 5. Conclusions

In this study, we systematically investigated the role of *GmSAUR46b*, a member of the *SAUR* gene family, in integrating light signals to regulate *G. max* growth and development. Using a combination of genetic manipulation (CRISPR/Cas9-mediated knockout and 35S-driven overexpression), transcriptomic profiling, anatomical characterization, and phenotypic analysis, we elucidated the functional mechanisms of *GmSAUR46b* in modulating specific agronomic traits under varying light conditions. Our findings demonstrate that *GmSAUR46b* functions as a key regulator in light-dependent developmental pathways. Its expression dynamically responds to light–dark transitions, indicating direct involvement in perceiving and transducing light signals. Transcriptomic analyses of SAMs, stems, and leaves across three developmental stages (V1, V2, V3) revealed that perturbation of *GmSAUR46b* significantly alters the expression of genes enriched in cell wall metabolism, hormone signaling (e.g., auxin and JA), and photosynthetic pathways. These results highlight the role of *GmSAUR46b* as a central hub integrating light cues with hormonal and cellular metabolic networks.

Anatomical and phenotypic analyses further confirmed the functional specificity of *GmSAUR46b*. Under shaded conditions, overexpression of *GmSAUR46b* led to a significant increase in leaf midrib thickness and stem trichome density compared to the WT plants, whereas the knockout lines exhibited reduced trichome density and diminished midrib thickness responses to shade. These findings indicate that *GmSAUR46b* specifically regulates leaf midrib development and stem trichome formation in a light-dependent manner—traits that are critical for mechanical support, vascular transport, and stress resistance. Additionally, the identification of numerous novel transcripts associated with stress resistance, hormone metabolism, and photosynthesis expands the known repertoire of soybean genes, providing new candidates for exploring *GmSAUR46b*-mediated regulatory networks. These transcripts, together with differentially expressed genes, underscore the complexity of the molecular pathways underlying *GmSAUR46b* function.

In summary, our study demonstrates that *GmSAUR46b* integrates light signals with hormonal and cell wall metabolic pathways to regulate soybean growth, specifically affecting leaf and stem traits. These findings deepen our understanding of the functional diversity of *SAUR* genes in soybean and offer valuable insights for molecular breeding strategies aimed at enhancing crop adaptability and yield under variable environmental conditions.

## Figures and Tables

**Table 1:** Results of new transcript types and quantities.

Code	Number
i	260
j	33,394
o	4204
u	3353
x	1543

Note: o represents the part on the same strand that overlaps with the reference exon; j represents at least one matching multi-exon; x represents the exon on the reverse strand that overlaps; i represents the intron that is completely contained within the reference transcript; u represents an unknown new transcript; all represents the total number of all types of new transcripts.

## Data Availability

The datasets used and/or analyzed in the current study are available from the corresponding author (Fengjie Yuan) on reasonable request.

## Supplementary Materials

The supporting information can be downloaded at https://www.mdpi.com/article/10.3390/ijms26189200/s1.
