# Supplementary material for: GmSAUR46b Integrates Light Signals to Regulate Leaf Midrib Thickness and Stem Trichome Density in Soybean
Source: Int J Mol Sci. 2025 Sep 20;26(18):9200. doi: 10.3390/ijms26189200 (PMC12471168; doi:10.3390/ijms26189200)
Supplement: Supplementary file 1 [file ijms-26-09200-s001.zip › Supplementary files/Supplementary figures.pdf]

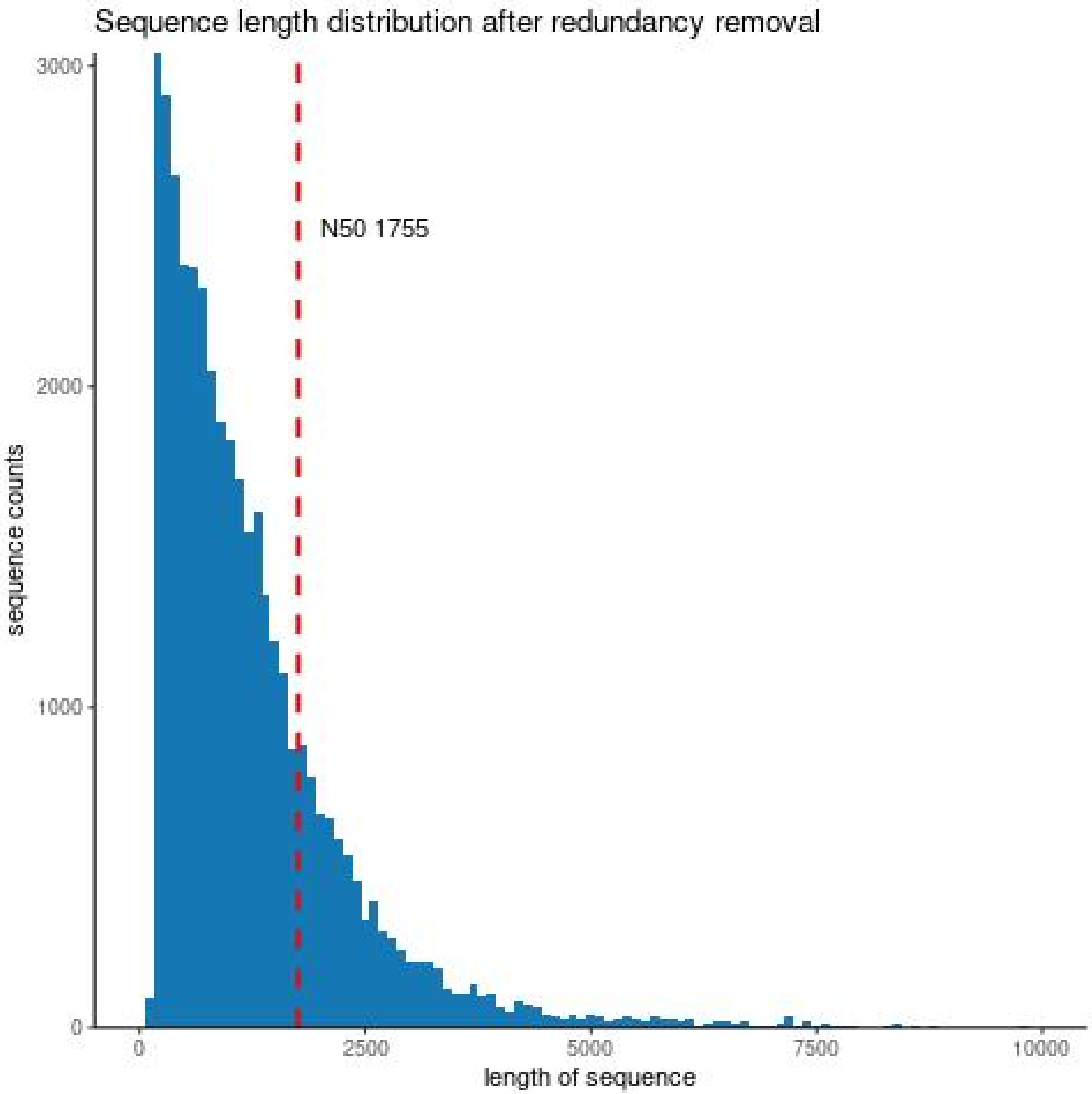

Figure S1. Distribution Chart of CDS Length of New Transcripts. The horizontal axis represents the length of the new transcripts; the vertical axis represents the number of new transcripts within each length range, with the red dotted line indicating the length of N50.

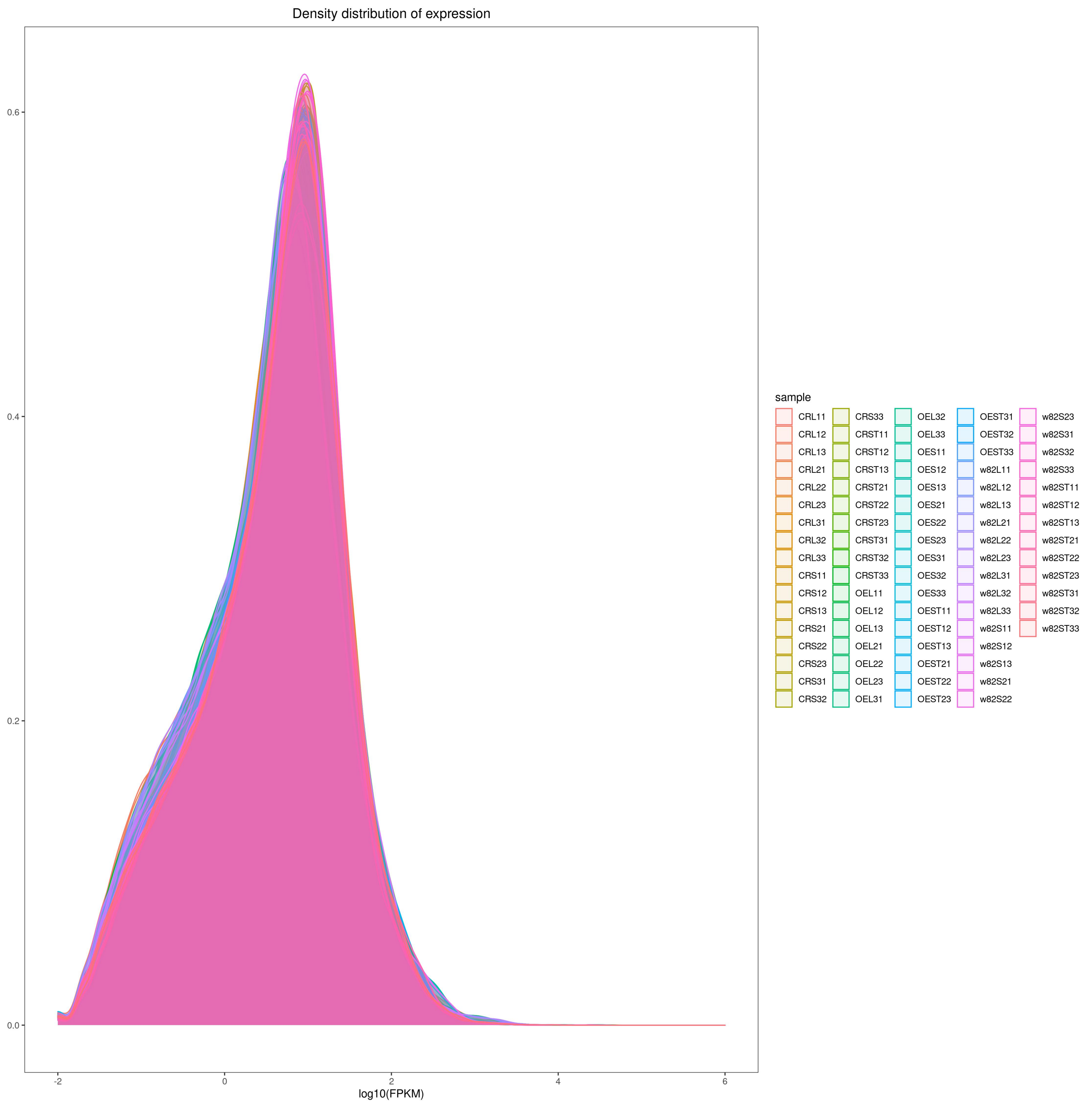

Figure S2. The FPKM density distribution graph shows a non-standard normal distribution. The area of each region is 1, indicating that the total probability is 1. The X-axis represents the logarithm of the expression level (FPKM value) to the base 10, and the Y-axis shows the density of genes with different expression level values. Different colors represent different samples.

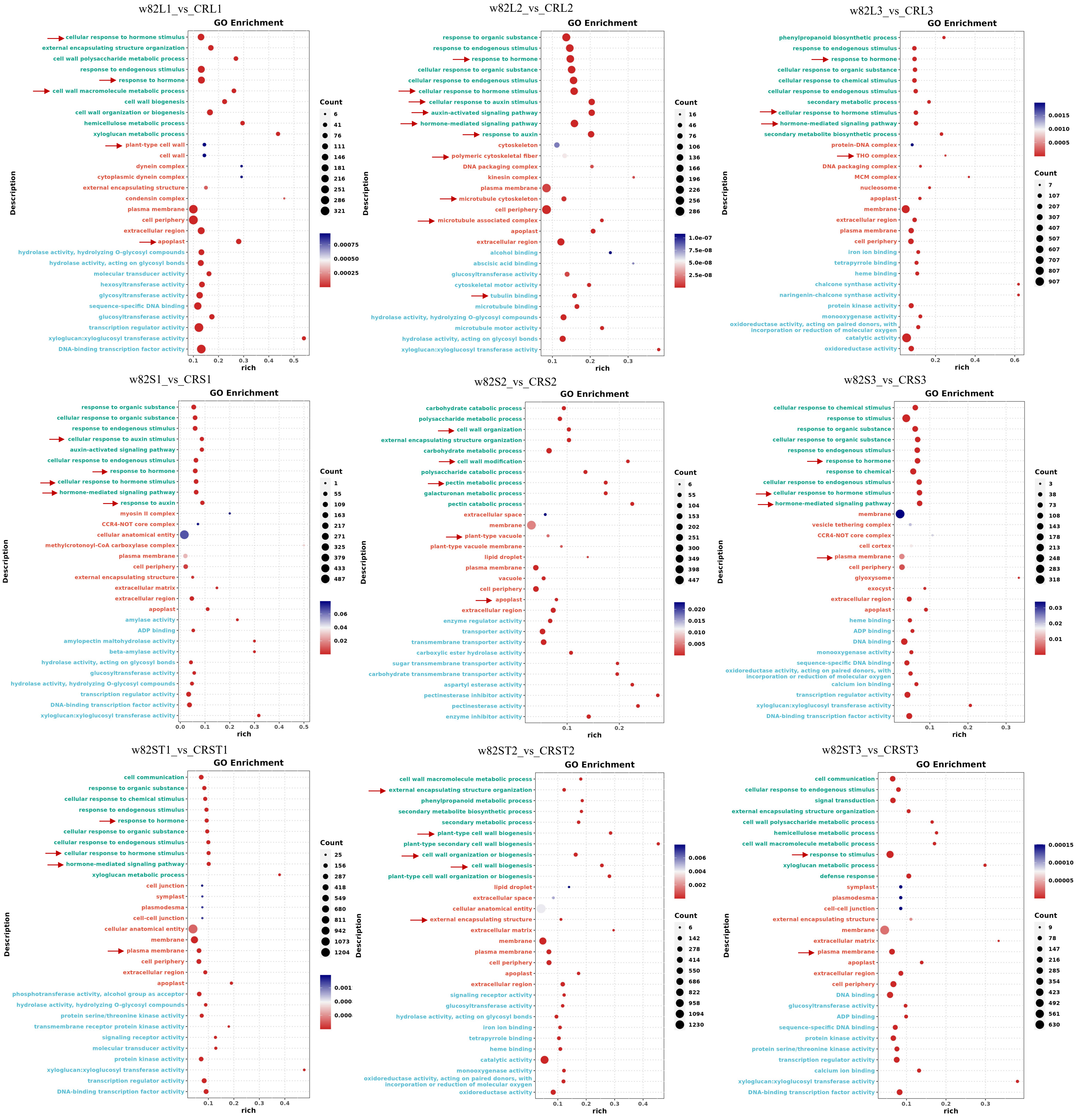
w82L1_vs_CRL1
GO Enrichment
cellular response to hormone stimulus
external encapsulating structure organization
cell wall polysaccharide metabolic process
response to endogenous stimulus
response to hormone
cell wall macromolecule metabolic process
cell wall biogenesis
cell wall organization or biogenesis
hemicellulose metabolic process
xyloglucan metabolic process
plant-type cell wall
cell wall
dynein complex
cytoplasmic dynein complex
external encapsulating structure
condensin complex
plasma membrane
cell periphery
extracellular region
apoplast
hydrolase activity, hydrolyzing O-glycosyl compounds
hydrolase activity, acting on glycosyl bonds
molecular transducer activity
hexosyltransferase activity
glycosyltransferase activity
sequence-specific DNA binding
glucosyltransferase activity
transcription regulator activity
xyloglucan:xyloglucosyl transferase activity
DNA-binding transcription factor activity
Description
rich
0.1 0.2 0.3 0.4 0.5
Count
6 41 76 111 146 181 216 251 286 321
0.00075 0.00050 0.00025
w82L2_vs_CRL2
GO Enrichment
response to organic substance
response to endogenous stimulus
response to hormone
cellular response to organic substance
cellular response to endogenous stimulus
cellular response to hormone stimulus
cellular response to auxin stimulus
auxin-activated signaling pathway
hormone-mediated signaling pathway
response to auxin
cytoskeleton
polymeric cytoskeletal fiber
DNA packaging complex
kinesin complex
plasma membrane
microtubule cytoskeleton
cell periphery
microtubule associated complex
apoplast
extracellular region
alcohol binding
abscisic acid binding
glucosyltransferase activity
cytoskeletal motor activity
tubulin binding
microtubule binding
hydrolase activity, hydrolyzing O-glycosyl compounds
microtubule motor activity
hydrolase activity, acting on glycosyl bonds
xyloglucan:xyloglucosyl transferase activity
Description
rich
0.1 0.2 0.3
Count
16 46 76 106 136 166 196 226 256 286
1.0e-07 7.5e-08 5.0e-08 2.5e-08
w82L3_vs_CRL3
GO Enrichment
phenylpropanoid biosynthetic process
response to endogenous stimulus
response to hormone
cellular response to organic substance
cellular response to chemical stimulus
cellular response to endogenous stimulus
secondary metabolic process
cellular response to hormone stimulus
hormone-mediated signaling pathway
secondary metabolite biosynthetic process
protein-DNA complex
THO complex
DNA packaging complex
MCM complex
nucleosome
apoplast
membrane
extracellular region
plasma membrane
cell periphery
iron ion binding
tetrapyrrole binding
heme binding
chalcone synthase activity
naringenin-chalcone synthase activity
protein kinase activity
monooxygenase activity
oxidoreductase activity, acting on paired donors, with incorporation or reduction of molecular oxygen
catalytic activity
oxidoreductase activity
Description
rich
0.2 0.4 0.6
0.0015 0.0010 0.0005
Count
7 107 207 307 407 507 607 707 807 907
w82S1_vs_CRS1
GO Enrichment
response to organic substance
cellular response to organic substance
response to endogenous stimulus
cellular response to auxin stimulus
auxin-activated signaling pathway
cellular response to endogenous stimulus
response to hormone
cellular response to hormone stimulus
hormone-mediated signaling pathway
response to auxin
myosin II complex
CCR4-NOT core complex
cellular anatomical entity
methylcrotonoyl-CoA carboxylase complex
plasma membrane
cell periphery
external encapsulating structure
extracellular matrix
extracellular region
apoplast
amylase activity
ADP binding
amylopectin maltohydrolase activity
beta-amylase activity
hydrolase activity, acting on glycosyl bonds
glucosyltransferase activity
hydrolase activity, hydrolyzing O-glycosyl compounds
transcription regulator activity
DNA-binding transcription factor activity
xyloglucan:xyloglucosyl transferase activity
Description
rich
0.0 0.1 0.2 0.3 0.4 0.5
Count
1 55 109 163 217 271 325 379 433 487
0.06 0.04 0.02
w82S2_vs_CRS2
GO Enrichment
carbohydrate catabolic process
polysaccharide metabolic process
cell wall organization
external encapsulating structure organization
carbohydrate metabolic process
cell wall modification
polysaccharide catabolic process
pectin metabolic process
galacturonan metabolic process
pectin catabolic process
extracellular space
membrane
plant-type vacuole
plant-type vacuole membrane
lipid droplet
plasma membrane
vacuole
cell periphery
apoplast
extracellular region
enzyme regulator activity
transporter activity
transmembrane transporter activity
carboxylic ester hydrolase activity
sugar transmembrane transporter activity
carbohydrate transmembrane transporter activity
aspartyl esterase activity
pectinesterase inhibitor activity
pectinesterase activity
enzyme inhibitor activity
Description
rich
0.1 0.2
Count
6 55 104 153 202 251 300 349 398 447
0.020 0.015 0.010 0.005
w82S3_vs_CRS3
GO Enrichment
cellular response to chemical stimulus
response to stimulus
response to organic substance
cellular response to organic substance
response to endogenous stimulus
response to hormone
response to chemical
cellular response to endogenous stimulus
cellular response to hormone stimulus
hormone-mediated signaling pathway
membrane
vesicle tethering complex
CCR4-NOT core complex
cell cortex
plasma membrane
cell periphery
glyoxysome
exocyst
extracellular region
apoplast
heme binding
ADP binding
DNA binding
monooxygenase activity
sequence-specific DNA binding
oxidoreductase activity, acting on paired donors, with incorporation or reduction of molecular oxygen
calcium ion binding
transcription regulator activity
xyloglucan:xyloglucosyl transferase activity
DNA-binding transcription factor activity
Description
rich
0.1 0.2 0.3
Count
3 38 73 108 143 178 213 248 283 318
0.03 0.02 0.01
w82ST1_vs_CRST1
GO Enrichment
cell communication
response to organic substance
cellular response to chemical stimulus
response to endogenous stimulus
response to hormone
cellular response to organic substance
cellular response to endogenous stimulus
cellular response to hormone stimulus
hormone-mediated signaling pathway
xyloglucan metabolic process
cell junction
symplast
plasmodesma
cell-cell junction
cellular anatomical entity
membrane
plasma membrane
cell periphery
extracellular region
apoplast
phosphotransferase activity, alcohol group as acceptor
hydrolase activity, hydrolyzing O-glycosyl compounds
protein serine/threonine kinase activity
transmembrane receptor protein kinase activity
signaling receptor activity
molecular transducer activity
protein kinase activity
xyloglucan:xyloglucosyl transferase activity
transcription regulator activity
DNA-binding transcription factor activity
Description
rich
0.1 0.2 0.3 0.4
Count
25 156 287 418 549 680 811 942 1073 1204
0.001 0.000 0.000
w82ST2_vs_CRST2
GO Enrichment
cell wall macromolecule metabolic process
external encapsulating structure organization
phenylpropanoid metabolic process
secondary metabolite biosynthetic process
secondary metabolic process
plant-type cell wall biogenesis
plant-type secondary cell wall biogenesis
cell wall organization or biogenesis
cell wall biogenesis
plant-type cell wall organization or biogenesis
lipid droplet
extracellular space
cellular anatomical entity
external encapsulating structure
extracellular matrix
membrane
plasma membrane
cell periphery
apoplast
extracellular region
signaling receptor activity
glucosyltransferase activity
hydrolase activity, acting on glycosyl bonds
iron ion binding
tetrapyrrole binding
heme binding
catalytic activity
monooxygenase activity
oxidoreductase activity, acting on paired donors, with incorporation or reduction of molecular oxygen
oxidoreductase activity
Description
rich
0.1 0.2 0.3 0.4
0.006 0.004 0.002
Count
6 142 278 414 550 686 822 958 1094 1230
w82ST3_vs_CRST3
GO Enrichment
cell communication
cellular response to endogenous stimulus
signal transduction
external encapsulating structure organization
cell wall polysaccharide metabolic process
hemicellulose metabolic process
cell wall macromolecule metabolic process
response to stimulus
xyloglucan metabolic process
defense response
symplast
plasmodesma
cell-cell junction
external encapsulating structure
membrane
extracellular matrix
plasma membrane
apoplast
extracellular region
cell periphery
DNA binding
glucosyltransferase activity
ADP binding
sequence-specific DNA binding
protein kinase activity
protein serine/threonine kinase activity
transcription regulator activity
calcium ion binding
xyloglucan:xyloglucosyl transferase activity
DNA-binding transcription factor activity
Description
rich
0.1 0.2 0.3
0.00015 0.00010 0.00005
Count
9 78 147 216 285 354 423 492 561 630

Figure S3. GO functional enrichment analysis of differentially expressed genes based on RNA-Seq sequencing results. W82, Williams 82; L, leaf; S, shoot apical meristem; ST, stem; 1, the stage when the first trifoliate leaf of soybean unfolds and flattens; 2, the stage when the second trifoliate leaf of soybean unfolds and flattens; 3, the stage when the third trifoliate leaf of soybean unfolds and flattens; the red arrow, the enriched pathway related to hormone metabolism; CR, the CRISPR/Cas9 gene edited mutants.

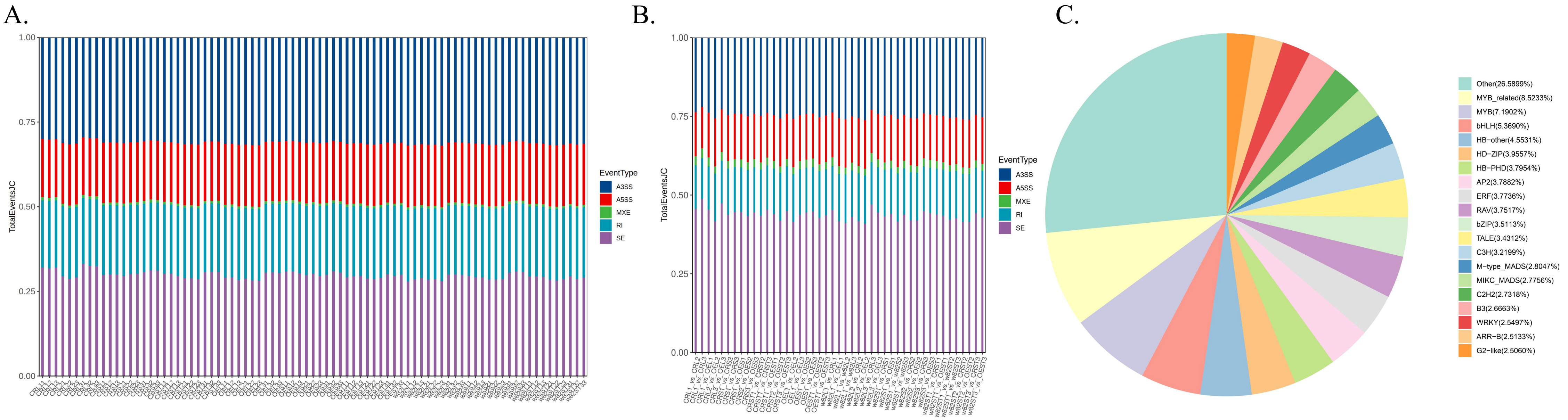

Figure S4. Analysis of alternative splicing and transcription factors in all comparison groups. Distribution map of all variable splicing event types in all samples (A); Distribution map of all comparison group variable splicing event types (B); Proportion chart of transcription factor families (C), and the different colors represent different families of transcription factors.

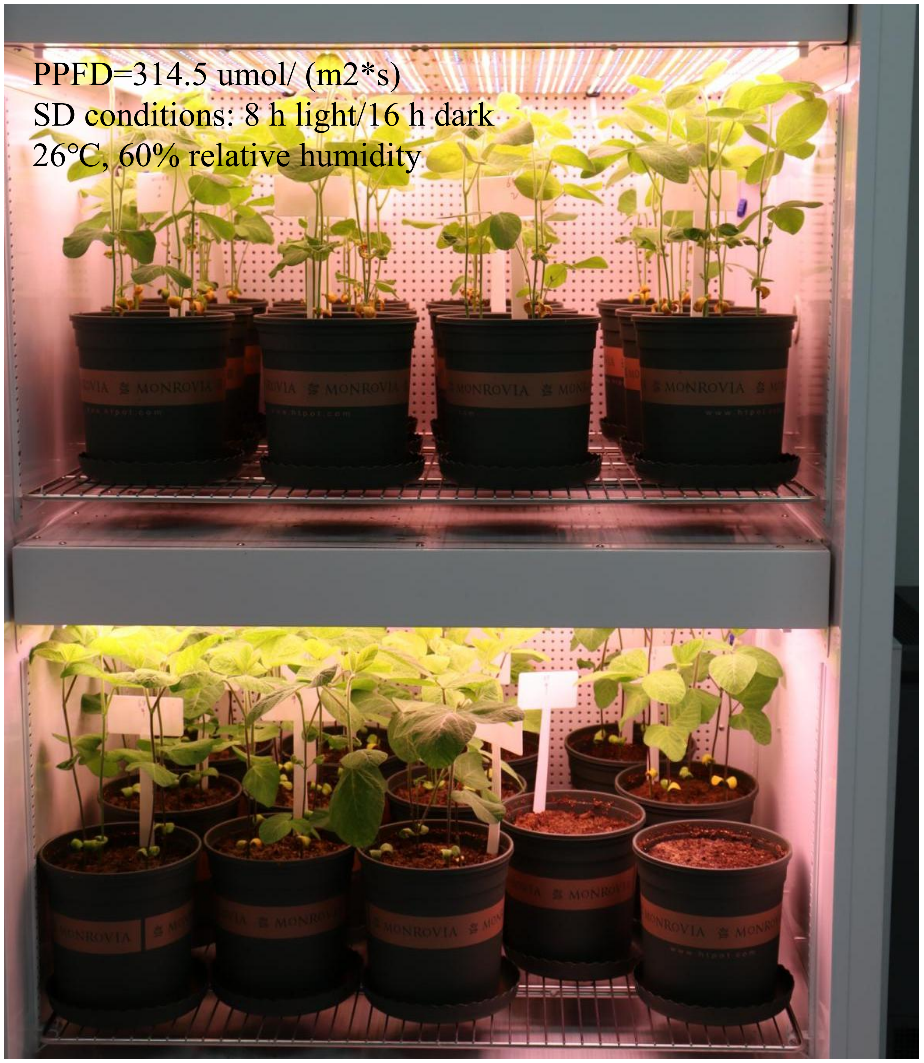

Figure S5. Growth conditions of soybean for the expression level analysis of light response. PPFD, photosynthetic photon flux density. SD, short day condition for 8 h light/16 h dark.
